# Recent trends in gene-targeted therapies and their influence on surgical decision-making in rheumatoid arthritis affecting the hands, feet, and ankles

**DOI:** 10.3389/fmed.2025.1742313

**Published:** 2026-01-12

**Authors:** Xiaolong Du, Xingxing Yu, Xuehai Ou, Deliang Cheng, Changming Zheng, Shaoyan Shi

**Affiliations:** 1 Honghui Hospital, Xi'an Jiaotong University, Xi'an, China; 2 Department of Laboratory Medicine, Xi'an Medical College, Xi'an, China

**Keywords:** autoimmune disorder, gene-targeted therapy, precision medicine, rheumatoid arthritis, surgical decision-making

## Abstract

Rheumatoid arthritis (RA) is an autoimmune disorder associated with chronic inflammation, progressive deformities of the joints, and limited mobility, especially in the hands, feet, and ankles. Recent discoveries in molecular genetics and immunotherapeutic approaches have enhanced the development of more effective RA medications. Gene-targeted therapy facilitates an accurate regulation of the immune system, ultimately preventing the destruction of joints. The new interventions that prevent the progression of the disease require orthopedic surgeons to reconsider the time and method of conducting surgery. This article discusses the latest developments in the field of gene-targeted therapy and their influence on the decision-making process in surgery. There has been a current change in clinical practice to involve early and preventive interventions for joint pathology to delay the use of surgery until it is unavoidable. These innovative techniques are based on the use of focused medicine and foster interdisciplinary cooperation of experts in orthopedics and rheumatology, which, in turn, increases the mobility of the patient and the overall quality of life.

## 1 Introduction

Rheumatoid arthritis results in cartilage degradation, joint deformity, and bone erosion. RA primarily affects the joints of the hands, feet, and ankles, causing joint pain, reduced mobility, and stiffness, which severely restricts daily activities (1). Treatment for RA has mainly aimed to relieve symptoms using nonsteroidal anti-inflammatory drugs (NSAIDs) and conventional disease-modifying antirheumatic drugs (DMARDs) such as methotrexate (2). However, treatment usually does not address the disease progression, and as a result, patients may still face long-term damage that could require reconstructive surgery (3).

Over the last 20 years, gene-based therapies have made a significant contribution to the field (4). Small-molecule agents and biologic therapeutics are now able to target disease processes at both cellular and genomic levels through the suppression of specific signaling cascades, such as interleukin 6 (IL-6), tumor necrosis factor 2 (TNF-2), and JAKs (5). These goal-oriented molecular therapies have resulted in significant remission durations, helped to maintain a joint architecture, and significantly reduced the need to undergo substantial surgical procedures. This has resulted in the restructuring of the time frame and performance of surgical operations.

Surgical interventions, which were suggested previously to recover severely affected joints, are currently performed as a regular procedure to improve functional results or correct mild deformities in patients with adequately controlled diseases (6, 7). The perioperative optimization of pharmacotherapy requires a collaborative approach by rheumatologists together with surgeons. This type of interdisciplinary coordination prevents the risk of infection and reduces the exacerbation of postoperative diseases.

This mini-review discusses recent developments in gene-targeted therapies for RA and evaluates the developments influencing surgical decisions for the hands, feet, and ankles. It highlights the coordination between molecular medicine and reconstructive surgery to help patients maintain mobility and independence.

## 2 Pathophysiology and genetic landscape of RA

RA is a complex autoimmune disease in which genetic factors, epigenetic mechanisms, and environmental influences come together to trigger ongoing inflammation of the synovial tissue and gradual joint damage (8, 9). This condition mainly affects the synovial lining, turning it into an aggressive pannus filled with macrophages, fibroblast-like synoviocytes, and activated T and B cells. This pathological development results in the overproduction of pro-inflammatory cytokines, destructive enzymes, and autoantibodies that damage the cartilage and bone (9, 10).

The human genome is susceptible to RA-related diseases through various loci and also determines the course of the disease progression (11). The most important genetic risk factor is the HLA-DRB1 gene and its shared epitope alleles, which regulate the antigen recognition ability of the immune system and facilitate the creation of autoantibodies. Other polymorphisms, like PTPN22, STAT4, TRAF1-C5, and CTLA4, are immunologically significant, leading to T-cell activity and cytokine signal transduction. The loci play a crucial role in sustaining immune homeostasis, T-cell activation, and cytokine generation (12). Resultantly, such genetic variations cause hyperactive immune cells that excessively secrete inflammatory mediators, including IL-1, IL-6, and TNF-*α*. Joint inflammation is maintained by the continued presence of an inflammatory milieu (8, 13–15).

**Figure 1:**
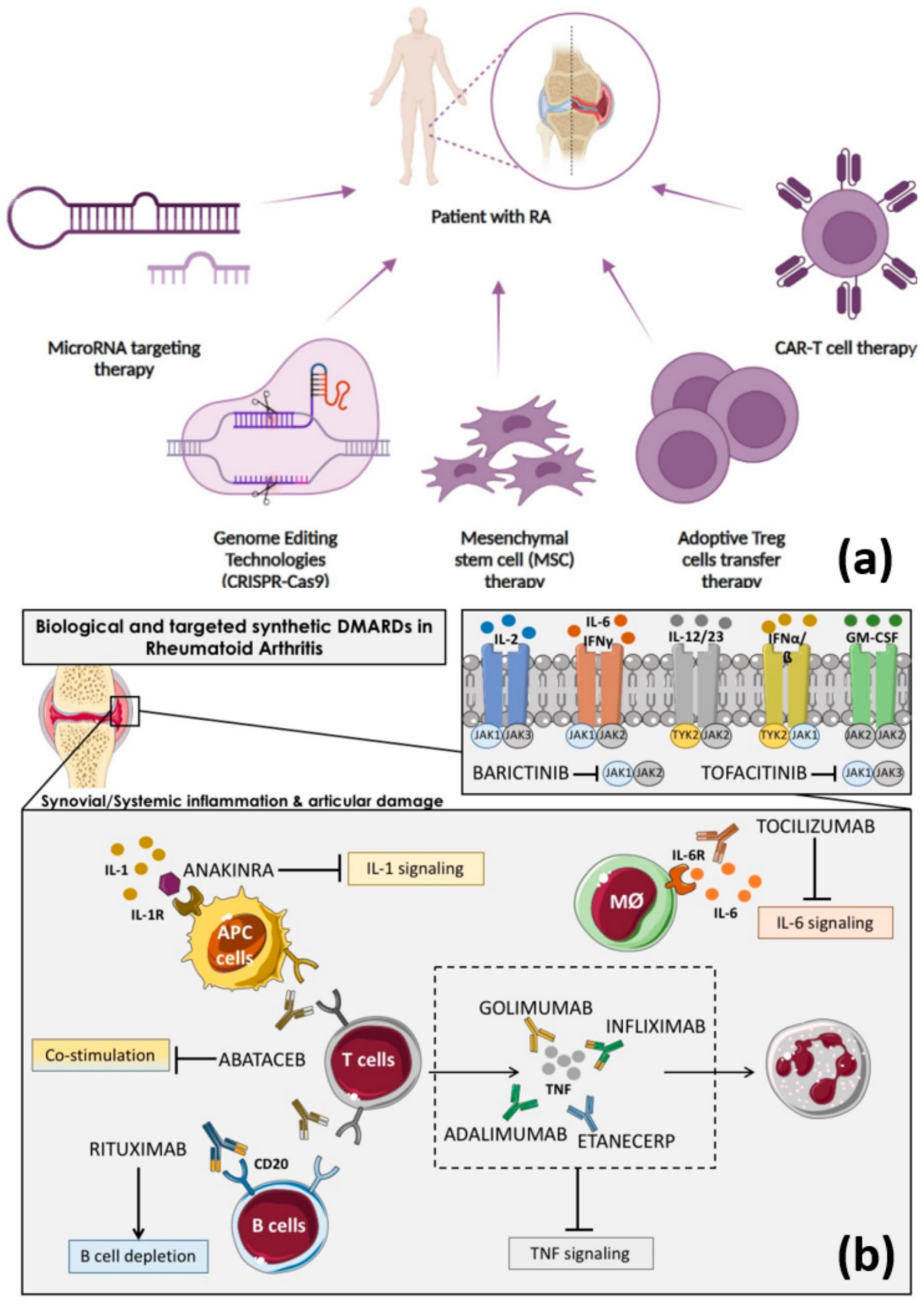
An overview of **(a)** cell-based therapies for treatment of RA, including genome editing and microRNA therapy (67), and **(b)** immunologic pathways targeted by the biological and targeted synthetic therapies in rheumatoid arthritis (39).

Genetic determinants can enhance the effect of epigenetic processes, such as histone modifications, microRNA activity, and DNA methylation. Excessive methylation of genes in synovial fibroblasts leads to increased cell growth and resistance to death, promoting the invasion of cartilage and bone by pannus. Certain microRNAs, such as miR-146a and miR-155, can regulate key cytokine signaling pathways and serve as biomarkers, as outlined in reference (16). The multifaceted nature of RA places the disease as one of the possible targets of gene-based therapeutic interventions (Figure 1a) (17).

RA can be triggered by the breakdown of the immune system, which is unable to tolerate the body tissues. CD4 + T cells in this pathogenic cascade stimulate B cells, which in turn synthesize autoantibodies including anti-citrullinated protein antibodies and rheumatoid factors (18, 19). The formation of immune complexes induces the attraction of inflammatory cells, the activation of complement pathways, and the release of reactive oxygen species and proteolytic enzymes, and causes damage to synovial tissue (20). With time, this may lead to erosive joint disease, deformities, and tears in tendons. The most common location of this pathology is the joints of the hands and feet, where the disease’s appearance is most intense.

Genetic etiology of RA has provided possibilities of developing more specific therapeutic agents (21). Such understandings of immune and signaling pathways have facilitated the production of new biologic and synthetic medications to inhibit TNF-alpha, JAKs, and IL-6 receptors. These treatments reduce joint destruction and inflammatory activities and thus are better compared to traditional ones (22). Recent molecular studies have been translated into actual clinical gains to patients with hand, foot, and ankle pathology (23). The prompt detection of genetic biomarkers by the specialist will allow predicting the disease course and avoiding the destruction of organs. Therefore, the successful reconstructive surgery demonstrate better chances to maintain the integrity of the joints in case they are treated with relevant pharmacologic interventions.

Advanced knowledge about RA genetics would not only provide the means of advancing the development of therapeutics but also allow surgeons with a sophisticated decision-making strategy. With this knowledge, clinicians will have an opportunity to determine the conditions of soft tissues, chronicity of the disease, and bone conditions regarding the molecular pathology of the disease (24). The introduction of genetic data into surgical planning opens a new era of individual treatment. This approach is a combination of immunogenetics and operative management, which improves procedures at the systemic and local surgical interfaces (25).

## 3 Evolution of gene-targeted therapeutics in RA

Gene-based therapies are an essential development in the treatment of autoimmune disorders (26). First-line treatment procedures mainly focused on the management of clinical symptoms and suppression of immune response with the use of NSAIDs and corticosteroids, and traditional DMARDs like sulfasalazine, methotrexate, and leflunomide (27). These drugs are beneficial in decreasing inflammation and preventing further destruction of the joint. However, they cannot target specific molecular pathways, often leading to systemic side effects and a failure to achieve remission. Advances in molecular biology and genomics have basically changed the nature of therapeutic methodologies and shifted the emphasis of approaches toward focused, molecule-specific therapies rather than generalized immunosuppression (28).

Biologic disease-modifying antirheumatic drugs (bDMARDs) are biosynthetic proteins that are designed to block inflammatory mediators in RA. The earliest bDMARDs, aimed at tumor necrosis factor-alpha (TNF-alpha), include infliximab, adalimumab, and etanercept. Clinical evidence has shown that these agents offer significant benefits by alleviating joint manifestations and slowing the rate of joint damage (29). These agents are specifically designed to target TNF-alpha, making cytokine blockade a viable therapeutic approach (30).

Current innovations have helped create new IL-1 and IL-6 antagonists. Sarilumab and tocilizumab, which are IL-6 receptor antagonists, are promising in patients not responsive to tumor necrosis factor (TNF) inhibitors (22). These agents interfere with the signaling pathways that control acute-phase responses, bone erosion mediated by osteoclasts, and B-cell differentiation. Additionally, anakinra IL-1 blockade has shown therapeutic effect in certain patients.

The second important innovation was targeted synthetic disease-specified antirheumatic drugs (tsDMARDs), specifically the group of Janus kinase inhibitors (JAK), tofacitinib, upadacitinib, and baricitinib. Extracellular cytokine biologics, such as JAK inhibitors, work in the cellular environment to regulate the JAK–STAT signaling cascade, a key component of cytokine-receptor interactions in the immune system (31). These small-molecule compounds are administered orally, resulting in improved clinical access. They act pharmacologically quickly, resulting in a wide array of suppressed inflammatory responses to multiple cytokine pathways.

At the same time, co-stimulatory modulation and B-cell depletion methodologies have increased the precision of treatment. A CTLA 4Ig fusion protein, Abatacept, blocks co-stimulation between T-cells or prevents CD80 and CD86 interaction with antigen-presenting cells. Rituximab, in contrast, targets CD20-positive B cells and, thus, neutralizes the production of autoantibodies and prevents the immune complexes formation (32). All these strategies help in countering both primary and downstream effector processes in the body, hence effective management of the disease.

Overall, these treatment procedures have triggered a significant transformational impact. They have considerably slowed down radiographic evolution, increased remission rates, and reduced the need for surgical intervention in severe cases of RA (33). Early treatment with accurate therapies can protect joint structure and function, particularly in the hands, feet, and ankles, where deformities are most common (34).

These therapeutic strategies enable the clinician to use genotypic information and biomarker (17) phenotypes in making therapeutic decisions (35). The use of such an integrative framework is effective in minimizing disease activity and providing information about the best time for surgical operations (36). Surgeons usually plan a reconstructive surgery when a patient demonstrates a stable immunologic status, which helps prevent perioperative complications. The innovations in the field of genomic editing and RNA-based therapeutics, such as the exploration of CRISPR-Cas9 and small interfering RNA (siRNA) mechanisms, show the potential of disease-modifying uses (Figure 1a) (26, 37). These are intervention-specific to immunogenetic etiologic processes, unlike symptomatic suppression. The introduction of gene-targeted biologics has significantly narrowed down individualized administration of RA and has increased the implementation of surgical processes into the standard lines of care (Figure 1b) (38, 39).

## 4 Influence on surgical decision-making for hand, foot, and ankle involvement

### 4.1 Decline in surgical frequency and shift in indications

Gene-targeted therapy has significantly minimized the need to undertake surgical intervention. Historically, synovectomy, tendon transfer, joint fusion, and arthroplasty were regularly used to treat the pain and deformity of the metacarpophalangeal and metatarsophalangeal joints (40). Nonetheless, biologic and targeted synthetic DMARDs are effective in suppressing synovitis and preventing structural damage, and thus enabling reversal or stabilization of early inflammatory changes prior to the emergence of irreversible damage. As a result, modern surgical practice currently focuses on the surgical treatment of functional deformities, including instability in the joint, tendon imbalance, and hypertrophy of the synovium (41).

### 4.2 Timing and preoperative optimization

Gene-targeted therapy has significantly transformed the timeline of surgical procedures (42, 43). In the modern clinical setting, operative surgery is now more and more planned during periods of clinical remission or with a dampened-down disease activity, reducing the risk of perioperative injury. Interdisciplinary collaboration between rheumatologists and surgeons is ineffective without each other, as biologic agents are typically used instead of surgery to reduce the risk of infection and maintain disease control, thereby preventing postoperative exacerbations (44). Pharmacokinetic processes inform this personalized periprocedural regimen of certain drugs; tumor necrosis factor inhibitors could be tapered off one dosing cycle before surgery, whereas Janus kinase inhibitors require a shorter period to cease therapy due to their high elimination kinetics (45).

### 4.3 Modification of surgical techniques

The recent developments in molecular treatment have played a role in the choice and expected results of surgical intervention (46). As part of the contemporary paradigm of observed hand deformities like swan-neck and ulnar drift, there has been a shift in the surgical paradigm to one that minimizes tissue disturbance, such as restoration of missing joint factors by means of surgical procedures like synovectomy, tendon repair, and open-ended reconstructive joint surgery. In the field of foot and ankle surgery, better control is achieved at the systemic level, which enables the timely correction action, such as metatarsal head resection or arthrodesis, which is performed before the appearance of the severe deformity (47). Biological modulation suppresses periarticular inflammation, enhances tissue integrity, and supports postoperative rehabilitation (48). This transition, therefore, promotes the maintenance of original joint structures and increases their overall functional ability.

### 4.4 Patient selection and risk assessment

The specific inclusion criteria of patients undergoing surgical intervention have been narrowed down through gene-targeted therapies. In preoperative evaluation, the variables exclusion, comorbid health conditions, and medication-related risks, e.g., cytopenia and delayed wound healing, are systematically examined (49). Additionally, auto-antibody titres and cytokine levels can also be used as automated biomarkers to predict the outcome of the surgery (50). In a situation where patients have shown a high disease activity despite the best biologic therapy, reconstructive surgeries may be necessary. On the other hand, patients responding to conservative management styles are those who have achieved molecular remission (51). This patient-oriented pattern highlights the importance of precision medicine in making informed decisions during surgery, where the benefits of intervention are crucial in the context of systemic safety.

## 5 Functional and clinical outcomes after gene-directed interference

### 5.1 Improvement in structural preservation and surgical success

Gene-targeted treatment patients often exhibit high-quality cartilage, increased bone densitometry, and better soft tissue integrity (52). These augmented tissue states result in a more desirable surgical environment, which subsequently minimises intraoperative complications and improves fixation stability. As an example, hand interventions, including tendon transfers or synovectomies, have a superior long-term response when using biologic therapy because of reduced recurrent synovitis and pannus proliferation. Similarly, foot and ankle resections, such as arthrodesis and osteotomy, are advantageous in terms of osteogenic healing and maintaining alignment, as systemic inflammation is well controlled (53).

### 5.2 Reduced postoperative inflammation and complication rates

Gene-targeted therapy reduces postoperative inflammatory flare-ups, which have been a significant challenge in the practice of RA surgery in the past (54). These therapies inhibit cytokine-mediated pathways, in particular, TNF-alpha and IL-6 signaling, which leads to decreased synovial hyperplasia and joint effusion and consequently leads to a more predictable postoperative course with fewer complications (1). Although biologic agents are postulated to be at a higher risk of infection, empirical evidence has shown that, when used at the right time, they are associated with equal to or lower rates of infection as compared to traditional immunosuppressants (55). Improved systemic control reduces the use of corticosteroids and, therefore, results in reduced complications associated with wound healing and tissue repair.

### 5.3 Functional and biomechanical outcomes

In a functional perspective, patients who have received gene-guided therapy exhibit a better range of movement, grip strength, and walking ability compared to those who received only conventional pharmacotherapy (56). Reconstruction of the hands, previously hindered by the disease’s persistent activity, is now recognized as a means to achieve long-term correction through increased dexterity and patient satisfaction. A partial fusion or soft-tissue balancing technique is also a joint-preservation method that is used in the foot and ankle, thus yielding more stable gait patterns and reducing painful movement (57). Proper management of inflammation also reduces the recurrence of progressive deformities, and patients can maintain postoperative gains on a longer basis.

### 5.4 Rehabilitation and long-term maintenance

The rehabilitation regimes used in postoperative evaluation have also changed to incorporate gene-targeted therapeutics (58). The regulation of inflammatory processes can promote the timely implementation of the treatment, and the simultaneous focus is made on the improvement of fine motor skills, muscular endurance, and mobility of the joints. A lesser need to spend some time in immobility decreases the stiffness of the joints and alleviates muscle atrophy, thus promoting recovery more quickly. Moreover, biological postoperative treatments help in supporting remission and increasing the effectiveness of the surgery. The integration of drug stability and a well-planned rehabilitative program lends more credence to the interdisciplinary integration that is important to sustained attention of the patient (59).

### 5.5 Integration of surgery within a gene-guided therapeutic framework

**Figure 2:**
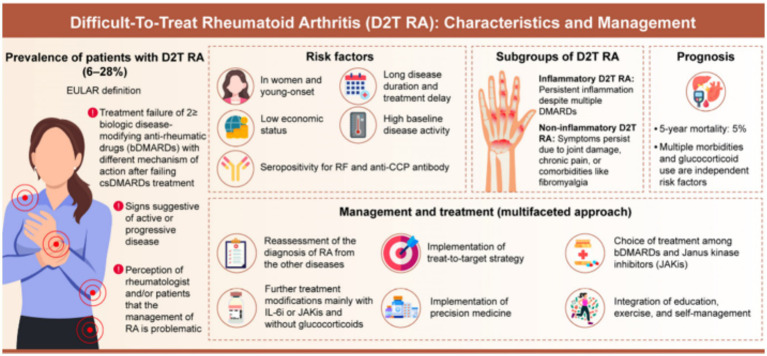
Schematic of gene-targeted therapy with surgical decision-making in RA (personalized approach and effective management) (17).

The collaboration between rheumatologists, geneticists, and surgeons helps to customize therapeutic programs that are in accordance with the unique molecular and functional disease peculiarities in the individual patient (60). It is a synergistic strategy involving systemic disease management and localized biomechanical changes, which will produce the best long-term results. The effectiveness of the model has been based on the combination of accurate pharmacologic treatment with the professional surgical practice, thus promoting a more holistic method of joint maintenance and overall shoulder rehabilitation (Figure 2) (39, 61).

## 6 Challenges and future directions

Although these developments have been made, several challenges still exist. Genetic variation has a considerable impact on the reaction of individuals to medications, and to predict the therapeutic results accurately, genomic profiling is necessary (62). Biologics are not available in under-resourced areas due to economic barriers, which leads to surgical intervention delays (63). Perioperative immunomodulation is a complex phenomenon to manage, as excessive reduction of immunity increases the risk of infection. Conversely, a rapid withdrawal can also trigger disease flares. Accordingly, standardized procedures during perioperative procedures are in high demand. In addition, the structural damage is observed in some patients despite biologic therapy, which highlights the need to use advanced therapeutic modalities, including gene silencing, CRISPR-based corrections, and RNA interference to induce remission (64).

Gene-targeted therapy in the future will be combined with regenerative medicine based on stem cells, or biomaterial scaffolds, to enable biologic reconstruction of joints (19). The adjunctive methodology will support precision surgery based on the use of the newest technologies of molecular diagnostics, multimodal imaging, and artificial intelligence (19, 65, 66). Remission maintenance and recovery improvement require continued learning, regular follow-ups, and multidisciplinary care. The general goal would be to move toward proactive disability prevention rather than reactive damage control through the combination of molecular therapeutics and reconstructive surgery.

## 7 Conclusion

The pattern of treatment of RA has experienced an essential change in line with the introduction of gene-targeted therapeutics, which have turned a pathology that was once fatal into one that can be managed over a long period. These procedures have rejuvenated a significant purpose of surgical intervention, the use of immunologic and genetic strategies, which reduce the frequency of operation and improve clinical outcome. Surgical procedures are combined with drug therapy to maintain the joint structure and to recover motor activity in patients that had attained a molecular remission. With the development of genetic profiling techniques, the combined use of gene-targeted therapies with regenerative surgery, and the broader use of biologic agents, the future of treatment is determined. Interdisciplinary teamwork of rheumatology, molecular biology, and reconstructive surgery is important. These developments will start a new era where the combination of accuracy in medicine and medical surgery ensures the maintenance of mobility and life quality in individuals with RA.
